# A Highly Immunogenic and Cross-Reactive Multi-Epitope Vaccine Candidate Against Duck Hepatitis A Virus: Immunoinformatics Design and Preliminary Experimental Validation

**DOI:** 10.3390/ijms262210958

**Published:** 2025-11-12

**Authors:** Yuanhe Yang, Xiaodong Chen, Anguo Liu, Jinxin He, Yunhe Cao, Pingli He

**Affiliations:** 1 State Key Laboratory of Animal Nutrition and Feeding, Frontiers Science Center for Molecular Design Breeding (MOE), China Agricultural University, Beijing 100193, China; yyh8695@163.com (Y.Y.); angelo524@foxmail.com (A.L.); caoyh@cau.edu.cn (Y.C.); 2 College of Veterinary Medicine, Shanxi Agricultural University, Jinzhong 030801, China; cxd1145275289@163.com (X.C.); hjx@sxau.edu.cn (J.H.)

**Keywords:** duck hepatitis A virus, multi-epitope vaccine, molecular docking, molecular dynamics simulation, prokaryotic expression

## Abstract

Duck viral hepatitis (DVH), a highly contagious disease, is caused primarily by duck hepatitis A virus (DHAV). The viral genotypes exhibit significant diversity, creating a challenge as monovalent vaccines fail to provide cross-genotype protection in ducklings. This study aimed to design a multi-epitope peptide vaccine targeting different genotypes of DHAV. Using immunoinformatics approaches, we systematically identified key antigenic determinants, including linear B-cell epitopes, cytotoxic T-cell epitopes (CTL), and helper T-cell epitopes (HTL). Based on these, a novel vaccine candidate was developed. The vaccine construct was subjected to rigorous computational validation: (1) Molecular docking with Toll-like receptors (TLRs) predicted immune interaction potential. (2) Molecular dynamics simulations assessed complex stability. (3) In silico cloning ensured prokaryotic expression feasibility. Then, we conducted preliminary experimental validation for the actual effect of the vaccine candidate, including recombinant protein expression in *E. coli*, enzyme-linked immunosorbent assay (ELISA) quantification of humoral responses, and Western blot analysis of cross-reactivity. ELISA results demonstrated that the vaccine candidate could induce high-titer antibodies in immunized animals, with potency reaching up to 1:128,000, and the immune serum showed strong reactivity with recombinant VP proteins. Western blot analysis using duck sera confirmed epitope conservancy across genotypes. Collectively, the multi-epitope vaccine candidate developed in this study represents a highly promising broad-spectrum strategy against DHAV. The robust humoral immunity it elicits, coupled with its demonstrated cross-reactivity, constitutes compelling proof-of-concept, laying a solid foundation for advancing to subsequent challenge trials and translational applications.

## 1. Introduction

Duck viral hepatitis (DVH) is a lethal infectious disease caused by duck hepatitis virus (DHV) [1] primarily affecting ducklings less than 3 weeks old, manifesting hepatomegaly and causing death within 3–7 days, with a mortality rate greater than 90% [2]. This poses substantial threats to global duck production. Three DHV serotypes (DHAV, DAstV-I, and DAstV-II) exhibit no cross-reactivity [1], with DHAV demonstrating the highest virulence [3]. Currently, DHAV genotypes (DHAV-1/2/3) show evolving epidemiology [3]: while DHAV-1 prevalence decreased through vaccination, DHAV-3 incidence and genotype co-infections (DHAV-1&3) have been rising [3,4], highlighting the need for cross-protective vaccines [5].

DHAV, the sole Avihepatovirus genus member [3], possesses a single-stranded positive-sense RNA genome containing a large open reading frame (ORF) and encoding an approximate 2200 amino acids VP polyprotein [6]. Proteolytic processing yields structural (VP0/VP1/VP3) and nine non-structural proteins [6,7]. The structural proteins harbor hypervariable regions critical for receptor binding, virulence, and immunogenicity [7]. Viral entry initiates through structural protein-mediated host cell attachment. Post-capsid disassembly, cytoplasmic RNA replication, and IRES-driven translation produce progeny virions that lyse host cells, perpetuating infection, ultimately causing host cell damage [8]. These features establish the DHAV structural proteins as prime vaccine targets.

Currently, there are no antiviral drugs that can effectively treat DHAV infections [9]. Previous studies have suggested that Chinese herbs, such as flavonoids [9], can block DHAV replication in ducklings, and locust flower polysaccharide (BSRPS) also exhibits anti-DHAV activity [10]. However, these methods have not been widely adopted. Passive or active immunization remains the primary means to reduce ducklings’ mortality. Passive immunization can provide immediate protection through administration of antibody-like substances to neutralize the virus, such as egg yolk antibodies (IgY) [3]. Nevertheless, passive immunity is limited by its short duration, requiring repeated immunization, and the high cost of IgY presents practical challenges. Consequently, active immunization using attenuated or inactivated vaccines is more widely accepted. Three attenuated DHAV-1 vaccines have been licensed in China [11], and in 2023, the attenuated DHAV-3 HB80 strain vaccine was officially approved [12]. All these vaccines offer effective prophylactic protection against corresponding genotype infections [11,12]. However, even within the same DHAV genotype, variations exist, and existing vaccines cannot provide complete protection [5,13]. To develop multivalent vaccines preventing infections from diverse DHAV strains, immunoinformatics approaches have emerged as a possible avenue.

Numerous multifunctional vaccine candidates have been developed using immunoinformatics tools, including those targeting SARS-CoV-2 [14], rabies virus [15], and alveolar echinococcosis (AE) [16]. Researchers have successfully expressed fusion proteins through prokaryotic, baculovirus, and lactic acid bacterial systems [17,18,19], with demonstrated in vivo efficacy. For instance, immunization of dwarf hamsters with a SARS-CoV-2 multi-epitope protein significantly reduced early-stage viral loads [19]. A *Senecavirus* A (SVA) multi-epitope recombinant protein conferred 80% homologous protection in piglets [17]. Furthermore, recombinant proteins derived from truncated VP1 [20] and VP3 [21] sequences not only stimulated antibody production in ducklings but also effectively blocked DHAV infection.

**Figure 1:**
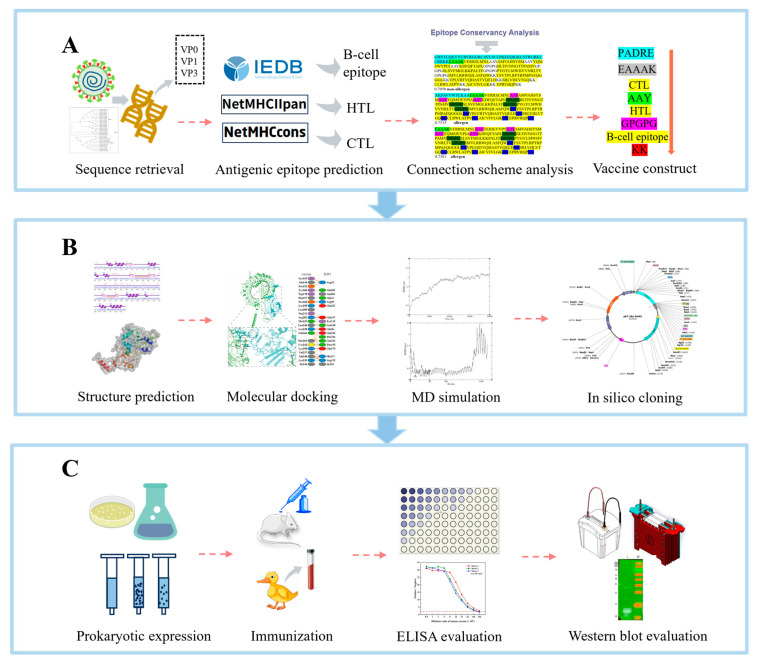
Schematic diagram of DHAV multi-epitope vaccine candidate design via immunoinformatics and initial experimental verification. (**A**) Preliminary construction of a multi-epitope vaccine candidate. (**B**) Bioinformatic validation of the multi-epitope vaccine candidate construction. (**C**) Preliminary experimental validation of the multi-epitope vaccine candidate bioactivity.

Building on these precedents, the present study employed immunoinformatics approaches to construct a multifunctional DHAV vaccine candidate. By predicting B-cell epitope, CTL, and HTL from three DHAV structural proteins [22], we designed a multi-epitope peptide vaccine candidate. Following the production of the recombinant protein via a prokaryotic expression system, we conducted preliminary in vitro and in vivo experiments that confirmed its high immunogenicity and cross-reactivity, thereby providing key preclinical evidence for subsequent vaccine efficacy evaluation (Figure 1).

## 2. Results and Discussion

### 2.1. Sequence Retrieval

The structural proteins (VP0, VP1, VP3) of DHAV are key determinants of viral virulence and immunogenicity [6]. For multi-epitope vaccine design, the initial step involves retrieving the protein sequences. It is noteworthy that extensive phylogenetic studies have demonstrated high sequence conservation within genotypes (intra-genotypic) for DHAV structural proteins. For instance, the intra-genotypic amino acid identity of the VP1 protein typically exceeds 92% [23,24,25,26]. This high degree of conservation implies that a vaccine designed based on representative strains within a DHAV genotype holds the potential to confer protection against the vast majority of circulating strains. Based on this premise, this study collected six DHAV structural protein sequences from the UniProt database (Table 1). These sequences were selected for their representation of the predominant DHAV genotypes (DHAV-1 and DHAV-3) circulating in the database, and to capture key antigenic variations between different genotypes while ensuring they are representative within their respective genotypes. Toll-like receptors (TLRs), as innate immune receptors, can detect pathogen-associated molecules directly or indirectly, thereby activating immune responses [27]. Research indicated that TLR-7 played a crucial role in triggering the innate immune response of ducks to DHAV infection and could serve as a critical receptor for evaluating vaccine efficacy [28]. Duck TLR7 (UniProt ID: U3I8Q9) was selected for subsequent immune validation of the multi-epitope vaccine. The VaxiJen server assessed the antigenicity of the sequences, all of which scored above 0.4, confirming their suitability as highly antigenic epitopes [29] for further analysis.

### 2.2. B-Cell Epitope Prediction

B cells are the primary mediators of humoral immunity. Predicting epitopes that bind to B-cell receptors and stimulate the immune system to produce specific antibodies is crucial for vaccine design [30]. This study utilized the IEDB server [22] to predict linear B-cell epitopes using three different methods. A threshold of 0.4 was applied, with higher scores indicating a greater likelihood of eliciting an immune response [29]. A total of seven highly antigenic epitopes were identified (Table 2), all with scores greater than 0.6.

### 2.3. Prediction of HTL Epitopes

HTL plays an important role in initiating specific immune responses upon recognition of foreign antigens, and it can utilize cytokines to facilitate humoral and cellular immunity to clear exogenous pathogens [31]. MHC molecules are essential in antigen recognition and presentation [32]. MHC class II molecules can bind with exogenous antigenic peptides to form complexes, which are then recognized by CD4+ T lymphocyte surface receptors (TCRs) located on HTL, thereby activating the immune response [32]. Upon activation, HTL secretes cytokines such as IL-2 and IFN-γ, which can promote HTL proliferation and differentiation, indirectly enhancing antibody production and promoting CTL function [32]. Therefore, predicting effective HTL epitopes is one of the critical strategies in vaccine design. In this study, five potentially antigenic HTL epitopes based on peptides binding to MHC alleles were predicted by the NetMHCIIpan server (Table 3). The epitopes showed high binding scores.

### 2.4. Prediction of CTL Epitopes

Similar to HTL, CTL is another crucial lymphocyte that mediates cellular immunity and plays a significant role in eliminating endogenous antigenic peptides [33]. CD8+ T cells belong to the CTL subpopulation. Upon recognition and binding of endogenous antigenic peptides with MHC class I molecules to form molecular complexes, these complexes are then recognized by CD8+ T cell surface receptors, thereby activating T cells [32]. CTLs can release effector molecules, such as perforin and granulysin, to lyse target cells [33], and they can also induce apoptosis by expressing high levels of FasL (the ligand for the target cell surface receptor Fas), which binds to target cells [34]. CTL epitopes contribute to the development of persistent cellular immunity [33]. Here, six potential CTL epitopes based on threshold IC50 values were screened using the NetMHCcons server (Table 4). All screened epitopes were assessed to be highly immunogenic.

### 2.5. Toxicity Prediction of the Selected Epitopes

Ensuring the safety and non-toxicity of selected epitopes is a prerequisite for vaccine candidate design. The ToxinPred online server, which employs machine learning-based models for batch toxicity prediction of peptides and is widely used for protein toxicity assessment [35]. The toxicity of seven linear B-cell epitopes, five HTL epitopes, and six CTL epitopes was predicted. The results indicated that all the selected sequences were non-toxic (Table 1, Table 2, Table 3 and Table 4), making them suitable for the design of candidate vaccines.

### 2.6. Cross-Genotype Conservation of Candidate Epitopes

To ensure the vaccine candidate can address the global genetic diversity of DHAV, we evaluated the conservation of its constituent epitopes. Analysis based on large-scale sequence datasets demonstrated the successful identification of multiple key epitopes with high conservation within their target genotypes (Table S1). Most notably, the epitope SEYAVTAMG was found to be exceptionally conserved (>95%) in both DHAV-1 and DHAV-3. This epitope serves as a rare cross-genotypic conserved target, forming the molecular cornerstone of the vaccine’s cross-protective capacity. The analysis also confirmed that the epitope EPVCFLN exhibits over 90% conservancy in both VP1 and VP3 proteins of DHAV-3, consistent with previously reported high intra-genotypic conservation [23,24,25,26]. Furthermore, several epitopes with substantial conservancy, such as SLSVFMGLKKPALFF (>85%), were identified in DHAV-1. These highly conserved epitopes, in combination with other selected epitopes of high immunogenicity, constitute the final vaccine sequence. This rational design aims to establish a broad foundation of protection through the conserved epitopes while simultaneously enhancing the overall immune response magnitude via highly immunogenic epitopes, thereby achieving a synergistic effect between these two strategies.

### 2.7. Designing Multi-Epitope Subunit Vaccine Candidate Construct

**Figure 2:**
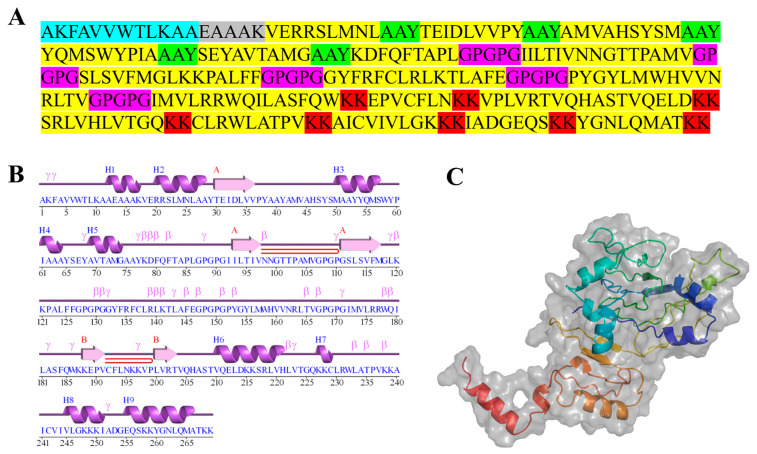
Primary sequence of DHAV vaccine candidate and prediction of secondary and Tertiary structure. (**A**) DHAV construct sequence. Epitopes are displayed in yellow; PADRE sequence is in blue, EAAAK linker is in gray, AAY linkers are shown in green, GPGPG linkers are highlighted with pink, and KK linkers are presented in red. (**B**) Secondary structure schematic of the DHAV vaccine candidate (PDBsum), showing α-helices (H1, H2…) as purple helical ribbons, β-strands (A, B…) as pink arrows, and random coils (with γ- and β-turns) as a purple line. (**C**) Tertiary structure prediction of DHAV vaccine candidate by Robetta and Galaxy Refine server.

Peptide vaccines usually exhibit poor immunogenicity, but their immunogenicity can be enhanced by connecting the selected epitopes with suitable linkers (Figure 2A) [36]. Linkers help maintain the flexibility and independence of amino acid residues, thereby facilitating proper protein conformation and enhancing the stability of epitope peptides. In this study, AAY, GPGPG, and KK linkers, which consist of flexible and hydrophilic amino acids, were selected to connect CTL epitopes, HTL epitopes, and B-cell epitopes, respectively. The PADRE sequence has been reported to enhance the stability of vaccine constructs and immune responses [36]. Therefore, it was incorporated at the N-terminus of the vaccine candidate using the rigid linker EAAAK to further promote the reactogenicity of the multi-epitope construct. The final vaccine construct comprised 269 amino acids, including one PADRE sequence, one EAAAK linker, five AAY linkers, five GPGPG linkers, and eight KK linkers.

### 2.8. Prediction of Antigenicity and Allergenicity

Immunogenicity, safety, and non-toxicity are the core issues in vaccine design. Therefore, the antigenicity and allergenicity of the multi-epitope vaccine candidate were evaluated using the VaxiJen and Allergen FP online servers, respectively. The antigenicity prediction threshold was set at 0.4, and the candidate achieved a score of 0.7514, indicating its potential to elicit a strong immune response. Allergic reactions may compromise both individual health and vaccine efficacy [35]. The constructed vaccine candidate was predicted to be non-allergenic, with its closest match being the human mitochondrial ATP-binding cassette transporter 10 (UniProtKB accession number Q9NRK6), a known non-allergenic protein. These computational findings suggest that the designed vaccine candidate is predicted to be safe and holds promise for being effective.

### 2.9. Prediction of Physicochemical Properties

The stability and biological function of the designed vaccine candidate were considered crucial factors. Therefore, the physicochemical properties of the vaccine were analyzed using the Expasy-ProtParam online tool. The results showed that the vaccine candidate consisted of 269 amino acid residues, with a molecular weight of 29.48 kDa and a theoretical isoelectric point of 9.92, indicating that it is an alkaline protein. The structure contains 12 negatively charged and 34 positively charged amino acid residues. Its instability index of 35.63 was lower than the threshold of 40 [37], suggesting the stable nature of the protein. The aliphatic index, reflecting the stability and antioxidant properties of the protein, was 88.51 for the obtained candidate, indicating good thermal stability and antioxidant capacity. Additionally, the GRAVY value, which assesses the protein’s hydrophilicity or hydrophobicity, was predicted to be 0.112, suggesting that the protein is hydrophobic (a negative score indicates a hydrophilic protein, whereas a positive score indicates a hydrophobic one) [37]. The hydrophilic KK linkers, in balance with the multiple core hydrophobic epitopes, may enhance the solubility of the protein. We also considered that these lysine-rich linkers could represent potential cleavage sites for trypsin; however, their strategic placement between epitopes ensures that any potential proteolysis would occur after immune recognition, thereby preserving the core immunogenicity of the candidate. The half-life of the structure in mammalian reticulocytes in vitro was estimated to be 4.4 h, while it was greater than 20 h in yeast and greater than 10 h in *E. coli*, demonstrating good stability in these heterologous expression systems.

### 2.10. Tertiary Structure Prediction

Accurate prediction of protein secondary structure serves as a crucial intermediate step in tertiary structure prediction, which is fundamental to understanding protein structure and function [35]. The predicted secondary structure states included α-helix, β-sheet, and random coil, which could influence vaccine candidate stability and function. To ensure the rationality of the tertiary structure, the secondary sequence was assembled and optimized based on allergenicity, antigenicity, and other factors, and the final model was predicted using PRABI. The results showed that the model was composed of 42.38% α-helix, 23.05% β-sheet, and 34.57% random coil. Sequences without known similar structures are more appropriately modeled from scratch [38]. Therefore, the Robetta online tools were utilized to perform de novo modeling of the protein sequence, followed by refinement of the initial model using the Galaxy Refine server. The model output files were all in PDB format, and the secondary (Figure 2B) and tertiary (Figure 2C) structures of the protein were visualized using PyMOL software.

### 2.11. High-Quality Tertiary Structure Model Validation

**Figure 3:**
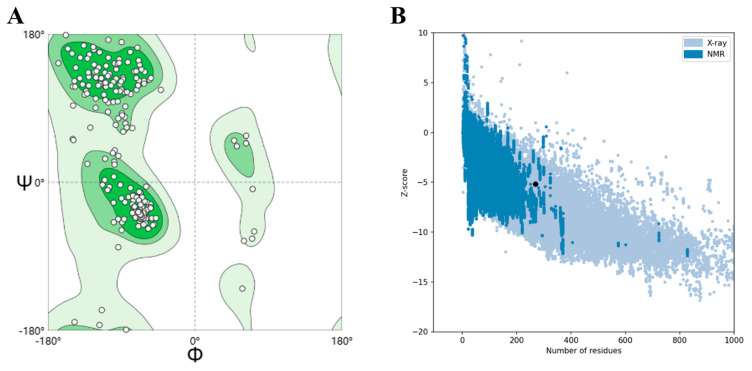
Validation of the modeled tertiary structure. (**A**) Ramachandran plot presenting the presence of amino acid residues in favored, allowed and outlier region. (**B**) The Z-score (−5.18) of the vaccine candidate model.

The structural model and its parameters were rigorously validated, providing a reliable theoretical basis for subsequent analysis of the vaccine candidate. The 3D structures of both initial and refined models were submitted to the SWISS-MODEL server for structural evaluation. The Ramachandran Plot reveals that approximately 86.89% of residues were in the allowed region before optimization, with about 2.25% in the outlier region. After optimization, about 91.57% of residues were in the allowed region, while approximately 0.80% were in the outlier region (Figure 3A). It is generally accepted that a model with over 90% of residues in the allowed region conforms to stereochemical rules [39]. Therefore, we considered the optimized model to have good quality and stability. Furthermore, the overall quality of the model was assessed using the ProSA-web server. The refined model achieved a Z-score of −5.18 (Figure 3B), which lies within the natural protein interval, indicating that the model is structurally sound and suitable for subsequent studies such as molecular docking.

### 2.12. Molecular Docking

**Figure 4:**
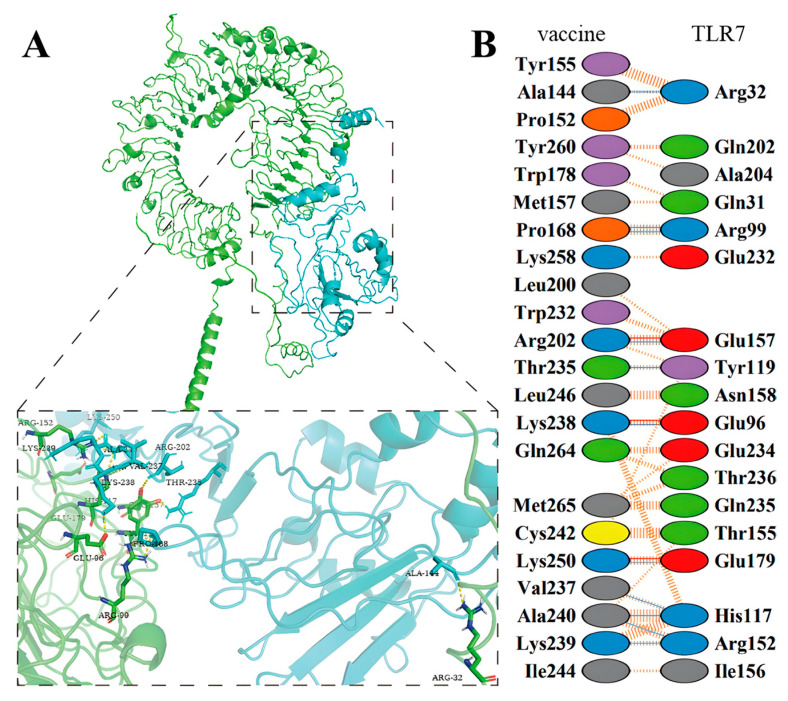
Molecular docking output of vaccine candidate and TLR-7 receptor obtained from PDBsum server represents the formation of stable complex. (**A**) Molecular docking overview of the multi-epitope vaccine candidate (cartoon) bound to the duck TLR7 receptor (surface). (**B**) Close-up view of the binding interface, highlighting key interacting residues.

The binding interaction between the optimized subunit vaccine candidate (ligand) and duck TLR7 (receptor; UniProt: U3I8Q9) was analyzed using HADDOCK with default parameters. The optimal model, selected based on a combination of low HADDOCK score, binding energy, and Z-score, exhibited characteristics of a stable complex. The HADDOCK score (−111.0 ± 21.2) falls within the typical range for stable protein–protein interactions [40]. A well-defined binding mode is indicated by the small cluster size (*n* = 2) and further supported by a low RMSD value of 8.9 ± 0.2 Å. Electrostatic forces were the predominant stabilizing factor for the interaction (−265.4 ± 51.6 kcal/mol), highlighting the critical role of charged residues. Furthermore, a Z-score of −1.8 suggests that the binding pose is native-like. Collectively, these results demonstrated that the designed vaccine candidate effectively recognizes duck TLR7, a key innate immune receptor, and suggest its potential to promote antigen presentation and immune activation (Figure 4). This model provides a solid foundation for further molecular dynamics (MD) simulations.

### 2.13. MD Simulation

**Figure 5:**
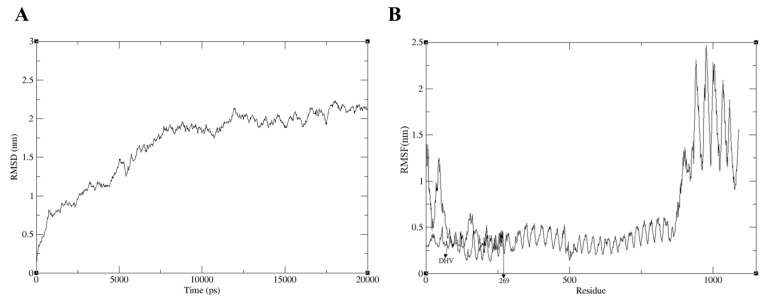
MD simulation of vaccine-receptor complex. (**A**) RMSD for the amino acid backbone of the complex, (**B**) RMSF of the amino acid’s side chains of the same.

The binding mode, conformational dynamics, and stability of the candidate-TLR7 complex were evaluated through a 20-ns MD simulation. Using GROMACS, we analyzed the molecular interactions and computed key dynamics metrics, including the Root Mean Square Deviation (RMSD), Root Mean Square Fluctuation (RMSF), and intermolecular hydrogen bonds. RMSD, which measures conformational drift from the initial structure [41], reached a plateau at 2.5 nm after 10 ns (Figure 5A), indicating that the system achieved equilibrium. RMSF, reflecting per-residue flexibility [41], showed low fluctuation (<1.5 nm) in the core interaction interface, while distinct peaks identified flexible residues elsewhere (Figure 5B). Hydrogen bonds play a critical role in protein-ligand recognition and structural stabilization [41]. The simulation revealed an average of 7 hydrogen bonds persistently stabilizing the complex. Collectively, these results suggest that the docked complex exhibits stable binding with limited conformational drift, a rigid binding interface, and a consistent network of hydrogen bonds.

### 2.14. In Silico Cloning into a Microbial Expression Vector

**Figure 6:**
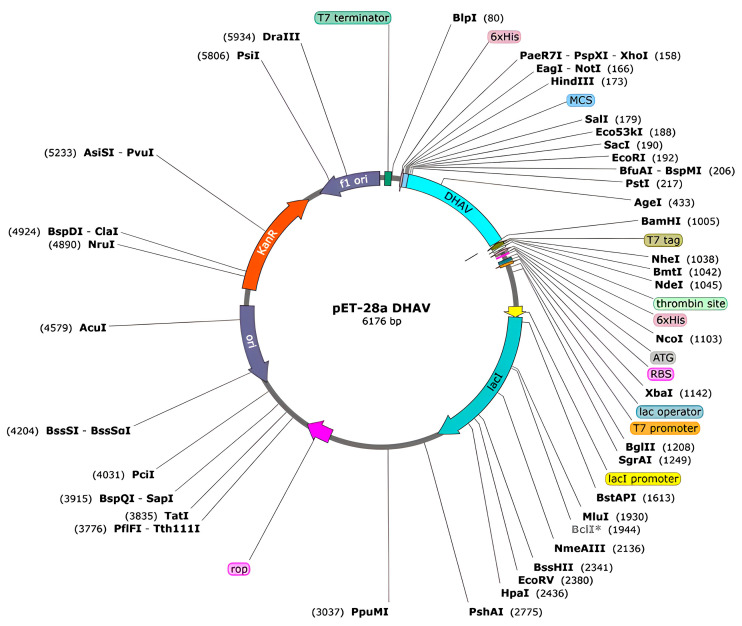
In silico cloning map showing the insert of the DHAV multi-epitope vaccine candidate protein.

The target gene was optimized based on the codon preference of *E. coli* before constructing the prokaryotic expression vector, resulting in a CAI of 0.85 and a GC content of 50.61%, both of which were within a reasonable range [42]. *Bam*H I and *Eco*R I restriction sites were introduced at the N-terminus and C-terminus of the sequence, respectively, for cloning into the multiple cloning site (MCS) of the pET-28a (+) vector, in which the His-Tag is small and does not interfere with the protein’s properties [43], facilitating protein detection and purification in experiments (Figure 6).

### 2.15. Expression and Verification of Subunit Vaccine Candidate

**Figure 7:**
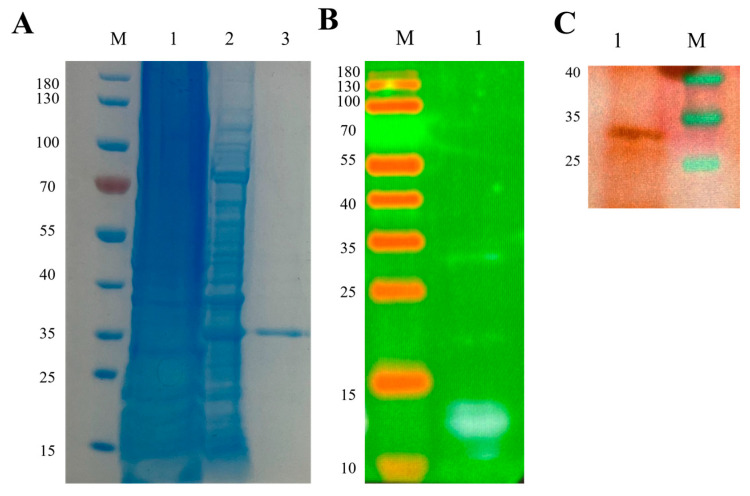
Prokaryotic expression and verification of the subunit vaccine candidate. (**A**) SDS-PAGE for protein expression verification; lane M: protein molecular weight marker, lane 1: cell lysate supernatant, lane 2: inclusion body pellet, lane 3: protein purification. (**B**) Western blot for protein expression verification using a monoclonal antibody against the His-tag; lane M: protein molecular weight marker; lane 1: vaccine protein. (**C**) Verification of vaccine candidate cross-reactivity by Western blot using positive duck serum infected with DHAV1 and DHAV3; lane 1: vaccine protein, lane M: protein molecular weight marker.

To achieve high-yield production, the target protein was induced during its logarithmic growth phase of *E. coli* [43]. SDS-PAGE results showed that a distinct band at approximately 30 kDa was observed solely in the induced group (Figure 7A), indicating successful expression, albeit primarily in the form of inclusion bodies. Western blot using an anti-His antibody further confirmed the identity of the expressed protein (Figure 7B). The inclusion bodies were solubilized using an equilibrium buffer containing 8 M urea [43], which fully exposed the His-tag. The target protein was then purified via nickel-affinity chromatography, with elution performed using a high-concentration imidazole solution. The purification efficiency exceeded 90%, as verified by SDS-PAGE. Finally, the purified protein was subjected to stepwise dialysis to remove the denaturant and facilitate refolding, resulting in a final concentrated protein solution of 0.5 mg/mL.

### 2.16. Induction of a High-Titer Antibody Response by the Vaccine Candidate

**Figure 8:**
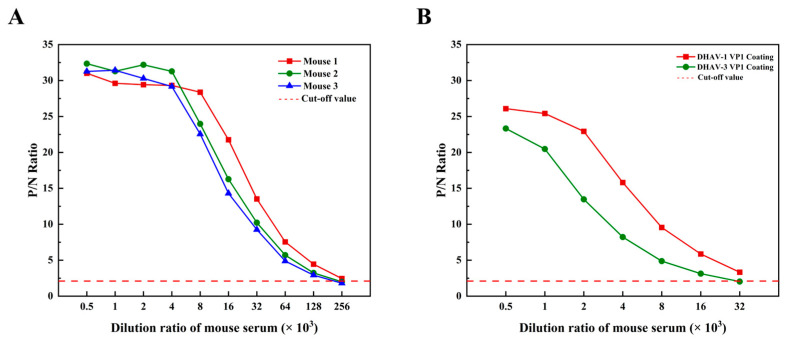
Serum titer after the last immunization in BALB/c mice. (**A**) Endpoint antibody titers in immunized mouse serum against the multi-epitope vaccine candidate. (**B**) Endpoint antibody titers in immunized mouse serum against recombinant VP1 protein. The P/N ratio was calculated as the OD450 value of the sample divided by the OD450 value of the negative control. The dashed line indicates the cut-off value (P/N ≥ 2.1). The endpoint titer was defined as the highest serum dilution that yielded a P/N ratio greater than or equal to the cut-off value.

To preliminarily verify the immunogenicity of the vaccine candidate under laboratory conditions, it was emulsified with Freund’s adjuvant and administered subcutaneously to mice. Serum samples were collected and analyzed after the four-immunization regimen. The results demonstrated a potent humoral immune response, with all immunized mice (*n* = 3) exhibiting antibody titers up to 1:128,000 (Figure 8A). This compelling experimental evidence confirms the excellent immunogenicity of the constructed vaccine, thereby corroborating our initial computational predictions.

It is important to note that these positive results must be considered within the context of the current study’s limitations. The high antibody titers reported here were achieved using a potent Freund’s adjuvant and a multiple-immunization protocol. This approach is standard in preclinical proof-of-concept research, aimed at rigorously evaluating the candidate vaccine’s maximum immunogenic potential under controlled laboratory conditions [44]. We fully acknowledge that this regimen presents limitations for practical commercial application in the duck industry. The robust immune response confirms that the construct functions as intended. However, translating this vaccine candidate into a commercially viable product for the poultry industry will require future work to formulate it with safe, approved veterinary adjuvants and to develop practical single-dose immunization strategies (e.g., in ovo or day-old immunization) to ensure its operational feasibility and effectiveness in field settings.

### 2.17. Serological Cross-Reactivity of the Vaccine Candidate Against DHAV Genotypes

The cross-reactivity of a vaccine candidate is a critical indicator for evaluating its potential broad-spectrum protection. To this end, this study innovatively employed serum from ducks co-infected with DHAV-1 and DHAV-3 as an assessment tool [45]. Western blot analysis confirmed specific binding between the vaccine antigen and positive sera (Figure 7C), demonstrating that the conformational epitopes within the candidate vaccine are accessible to polyclonal antibodies induced by natural infection. This further indicates the successful preservation of key immunodominant epitopes in our design. The cross-reactive antibody response was quantitatively assessed by indirect ELISA (Figure 8B). The immune serum exhibited high endpoint titers against recombinant VP1 proteins from both DHAV-1 (1:32,000) and DHAV-3 (1:16,000), demonstrating a substantial cross-reactivity index. These findings provide direct evidence for the rational vaccine design. Of particular importance, the synergistic integration of epitope conservancy analysis, TLR7 binding experiments (Figure 4), binding assay of mouse serum to VP recombinant proteins, and cross-reactivity data with duck serum has enabled the construction of a multi-tiered evidence chain encompassing computational prediction, molecular interaction, and serological verification.

This study remains in the early stages of vaccine development, with the current work marking a successful transition from conceptual design to preclinical validation. It must be noted that while this research has established the candidate vaccine’s capacity to elicit a potent cross-reactive humoral immune response, it has not yet addressed the final protective efficacy in the natural host (ducks) nor elucidated the contribution of T-cell immunity to protection. Although the high antibody titers in the murine model, the cross-reactivity of immune serum with VP proteins, and the cross-reactivity of the candidate with sera from co-infected ducks provide strong support for the proof-of-concept, these immunological parameters do not directly equate to protective capability against a live viral challenge. Therefore, the central task for subsequent research is to confirm its protective effect through standardized challenge models in ducks, to delve into the mechanisms by which it elicits a comprehensive immune response, to explore practical immunization strategies capable of inducing effective immune protection, and thereby to lay the foundation for ultimate application.

## 3. Methodology

### 3.1. DHAV Protein Sequence Retrieval

Firstly, amino acid sequences for the three DHAV structural proteins (VP0, VP1, VP3) and duck Toll-like receptor 7 (TLR7; UniProt ID: U3I8Q9) were obtained from the UniProt database (https://www.uniprot.org/) (accessed on 10 July 2024) and stored in FASTA format. To support subsequent epitope prediction for subunit vaccine candidate design, the antigenic potential of these sequences was evaluated using the VaxiJen server (https://ddg-pharmfac.net/vaxijen/VaxiJen/VaxiJen.html) (accessed on 16 July 2024) [29].

### 3.2. Prediction of Linear B-Cell Epitopes

Three distinct computational methods from the Immune Epitope Database (IEDB; https://tools.iedb.org/bcell/) (accessed on 17 July 2024) [22] were employed to systematically predict linear B-cell epitopes in the multi-epitope protein. The BepiPred Linear Epitope Prediction 2.0 web server utilizes a random forest algorithm to identify epitopes through antibody-antigen structural analysis, demonstrating enhanced predictive accuracy compared to conventional approaches [22,46]. The Kolaskar & Tongaonkar Antigenicity method evaluates antigenic potential by analyzing amino acid physicochemical properties, providing insights into the target protein’s immunogenic recognizability [46]. As hydrophilic regions frequently mediate antigen-antibody interactions [30], the Parker Hydrophilicity Prediction tool was implemented to map potential epitope regions through hydrophilicity distribution analysis. Consensus epitope sequences were derived through integration of results from all three methodologies. Antigenicity validation was performed using the VaxiJen server, with epitopes scoring more than 0.4 classified as highly antigenic [29]. Potential allergenic properties were assessed through the Allergen FP v2.0 server (http://ddg-pharmfac.net/AllergenFP/) (accessed on 25 July 2024) [35].

### 3.3. Prediction of Helper T-Cell Epitopes

Helper T Lymphocyte (HTL) epitopes were predicted using the NetMHCIIpan3.1 server (http://www.cbs.dtu.dk/services/NetMHCIIpan-3.1/) (accessed on 17 July 2024) [31]. Peptide length was set to 15 amino acids, and the server predicted MHC allele-binding peptides. Predicted peptides were assigned percentile ranking: peptides in the top 0.5% were classified as strong binding epitopes, while those in the top 2% were designated weak-binding epitopes. All peptides within the top 2% percentile were selected for further analysis. Antigenicity was assessed using the VaxiJen server [29], with a threshold > 0.4 defining high-antigenicity epitopes. Allergenicity was evaluated via the Allergen FP v.2.0 server (accessed on 25 July 2024) [35].

### 3.4. Prediction of Cytotoxic T-Lymphocyte Epitopes

The NetMHCcons 1.1 server (http://www.cbs.dtu.dk/services/NetMHCcons) (accessed on 18 July 2024) was used for predicting Cytotoxic T Lymphocyte (CTL) epitopes [33]. Epitope length was fixed at 9 amino acids, with screening based on half maximal inhibitory concentration (IC_50_) threshold values: IC_50_ < 50 nM indicates strong binders, while IC_50_ > 500 nM indicates weak binders. The percentile rank was set to 0.5 for strong binders and 2 for weak binders. The top two epitopes by percentage ranking were selected for antigenicity analysis using the VaxiJen server, with scores exceeding 0.4 defined as high antigenic epitopes [29]. Allergenicity assessment was performed using the Allergen FP v2.0 platform (accessed on 25 July 2024) [35].

### 3.5. Prediction of Toxic Immunogenicity of Cellular Epitopes

To evaluate the potential toxicity of the predicted peptide sequences, the ToxinPred online server (https://webs.iiitd.edu.in/raghava/toxinpred/) (accessed on 28 July 2024) was utilized to assess the safety of all screened linear B-cell epitopes, HTL, and CTL epitopes [31,33,35].

### 3.6. Epitope Conservancy Analysis

To evaluate the cross-protective potential of the vaccine candidate, conservancy analysis was performed on all selected epitopes prior to vaccine sequence assembly. Structural protein sequences of DHAV-1 and DHAV-3 were retrieved from the NCBI database. To ensure robust and statistically significant conclusions, the analysis was primarily based on the most representative datasets, namely DHAV-1 VP1, DHAV-3 VP1, and DHAV-3 VP3. Conservancy calculation was conducted using the IEDB Conservancy Analysis tool (accessed on 29 July 2024).

### 3.7. Design of a Candidate Multi-Epitope Subunit Vaccine

The multi-epitope subunit vaccine candidate was constructed by fusing the selected CTL, HTL, and B-cell epitopes using appropriate linkers. These linkers preserve the structural flexibility of amino acid residues while facilitating optimal conformational arrangements of the vaccine protein. CTL epitopes were connected via an AAY linker, specifically designed to create TAP-binding motifs and enhance epitope presentation. HTL epitopes were conjugated using GPGPG spacers to promote HTL responses and maintain conformation-dependent antigenicity. B-cell epitopes were integrated through KK connectors. To augment vaccine candidate immunogenicity, a PADRE peptide adjuvant was incorporated at the N-terminus via an EAAAK linker [36], yielding the final multi-epitope vaccine candidate construct.

### 3.8. Prediction of Antigenicity and Allergenicity of the Designed Subunit Vaccine Candidate

The antigenic potential of the designed subunit vaccine candidate was evaluated using the VaxiJen server (accessed on 3 August 2024). This computational tool applies machine learning algorithms to analyze physicochemical properties of known antigenic sequences for predicting the antigenic potential of protein-based vaccines [29]. Using the default threshold of 0.4 for bacterial vaccine prediction, sequences achieving scores above this value were considered probable immunogens [29]. The safety assessment was performed through allergenicity prediction using the Allergen FP v2.0 server (accessed on 3 August 2024) [35].

### 3.9. Prediction of Various Physicochemical Properties

Physicochemical properties were analyzed using the Expasy-ProtParam online tool (http://web.expasy.org/protparam/) (accessed on 4 August 2024). Based on the amino acid composition of the protein, physicochemical parameters were calculated using this tool [37]. We determined the molecular weight of the multi-epitope vaccine candidate protein and calculated its isoelectric point using amino acid pKa values. Protein stability was predicted based on the ratio of stable to unstable amino acids, with sequences scoring below the established threshold of 40 being judged as stable [37]. Additionally, the half-life, aliphatic index, and grand average hydropathicity (GRAVY) of the protein could also be predicted.

### 3.10. Prediction of Tertiary Structure

To gain a deeper understanding of the physicochemical properties, structural dynamics, and function of the protein, the secondary structure was predicted using the PRABI web server (https://npsa-prabi.ibcp.fr/cgi-bin/npsa_automat.pl?page=npsa_gor4.html) (accessed on 10 August 2024) [47] to quantify the relative proportions of different secondary structure elements. Subsequently, ab initio modeling was performed using the Robetta server (https://robetta.bakerlab.org/) (accessed on 11 August 2024) to predict the tertiary structure [38]. Following initial modeling, the predicted three-dimensional (3D) structure was refined through the Galaxy Refine server (http://galaxy.seoklab.org/cgi-bin/submit.cgi?type=REFINE) (accessed on 15 August 2024) employing a molecular dynamics (MD) simulation algorithm [48].

### 3.11. Optimization and Verification of Tertiary Structure

To ensure the accuracy and reliability of the established model, validation of its tertiary structure is crucial. This was achieved through Ramachandran Plot and Z-Score Plot analyses using the SWISS-MODEL server (https://swissmodel.expasy.org) (accessed on 19 August 2024) [35] and ProSA-web (https://www.came.sbg.ac.at/prosa.php) (accessed on 21 August 2024), respectively [39]. The Ramachandran plot visualizes the dihedral angles (φ and ψ) of backbone residues in the multi-epitope protein structure, revealing whether the conformations defined by dihedral angle pairs for adjacent peptide units fell within permissible regions, thereby assessing the stereochemical properties of the protein [39]. The Z-score plot evaluated whether the multi-epitope protein fell within the score range of similarly sized natural proteins, providing an assessment of the overall quality of the model [39].

### 3.12. Protein–Protein Docking

The HADDOCK server (https://wenmr.science.uu.nl/haddock2.4/) (accessed on 25 August 2024)was employed to model the interaction forces between the multi-epitope subunit vaccine candidate and its receptor [49], which involved performing molecular docking of the ligand and receptor to predict their optimal binding mode [50]. The ligand was the optimized multi-epitope protein, while the receptor was selected as duck TLR7 (UniProt: U3I8Q9). The server utilized protein information using Ambiguous Interaction Restraints (AIRs) to drive the docking process. Multiple independent docking simulations were performed to assess the interactions, predict binding modes and affinities, and ensure that the overall binding free energy of the complex was minimized. The results of the protein–protein complex docking were presented via the online PDBsum server (https://www.ebi.ac.uk/thornton-srv/databases/pdbsum/) (accessed on 27 August 2024) and were visually analyzed and interpreted using the PyMOL (version 2.5.0, Schrödinger, LLC, New York, NY, USA) software [41].

### 3.13. Molecular Dynamics Simulation

Molecular dynamics (MD) simulation was utilized to investigate the dynamic motions, binding patterns, and structural stability of candidate constructs at the atomic level. The MD simulations of molecular docking complexes were conducted using GROMACS (version 2024.2, Royal Institute of Technology, Stockholm, Sweden) [51]. During pre-processing, the coordinate and topology files of the simulated complex were generated, and the simulation system was constructed. The latter was configured as follows: the optimal conformation of the complex was placed in a cubic box filled with SPC water model under the AMBER99 force field, maintaining a 10 Å buffer between the complex and box boundaries, and Na^+^ and Cl^−^ were added to neutralize the system charge.

During the simulation process, the steepest descent algorithm was first employed to minimize the system energy, thereby eliminating unfavorable contacts and collisions among atoms and avoiding errors in the simulation caused by unreasonable structures. Then, NVT equilibration was conducted to bring the system temperature to the desired value and keep it at a basically constant level, followed by NPT equilibration to ensure the number of particles and pressure in the system remained constant. The final MD simulations utilized a 2 fs time step for integration and a 1 ps constant for pressure coupling. Following system equilibration, a 20-ns production phase was executed, saving atomic coordinates at 10 ps intervals. Resultant trajectories underwent analysis via root mean square deviation (RMSD) and root mean square fluctuation (RMSF) metrics [41].

### 3.14. Codon Optimization and Computational Cloning Simulation

To validate the structural accessibility of the subunit vaccine candidate construct and experimental reliability, computational cloning was performed using SnapGene (version 6.0.2, GSL Biotech LLC, Chicago, IL, USA) software to insert the gene sequence into the prokaryotic expression vector pET-28a (+). First, the amino acid sequence was reverse-translated into a nucleotide sequence using an online reverse translation tool (https://www.novopro.cn/tools/rev_trans.html) (accessed on 25 September 2024). Subsequently, codon optimization was conducted through the GenScript Rare Codon Analysis server (https://www.genscript.com/tools/rare-codon-analysis) (accessed on 25 September 2024) according to *E. coli* codon usage, with optimization of Codon Adaptation Index (CAI) and GC content [42]. Following this, NEBcutter (http://nc2.neb.com/NEBcutter2/) (accessed on 26 September 2024) was employed to screen for potential commercial restriction sites within the sequence [42]. Restriction sites corresponding to *Bam*H I and *Eco*R I were engineered at the 5′ and 3′ termini, respectively, and the final optimized sequence was ligated into the pET-28a (+) expression vector.

### 3.15. Prokaryotic Expression and Verification of Multi-Epitope Subunit Vaccine Candidate

Prokaryotic expression utilized the *E. coli* BL21(DE3) strain (TransGen Biotech Co., Ltd., Beijing, China) [43]. Beijing TsingKe Biotechnology Co., Ltd. (Beijing, China) was commissioned to synthesize and clone the gene into the prokaryotic expression vector pET-28a (+), with the plasmid subsequently transformed into the *Top10* strain. The *Top10* strain (TransGen Biotech Co., Ltd., Beijing, China) was streaked onto an LB agar plate containing 1 mg/mL kanamycin (Kan^+^) (Solarbio Science & Technology Co., Ltd., Beijing, China) and incubated overnight. The next day, a single colony was transferred into LB liquid medium containing 1 mg/mL Kan^+^, then cultured in a shaking incubator at 37 °C and 220 rpm overnight. The activated seed culture was transferred at a 1:100 ratio into 100 mL of LB-Kan^+^ liquid medium until reaching an OD_600_ of 0.6–0.8, and 1 mM IPTG (Solarbio Science & Technology Co., Ltd., Beijing, China) was added for induction, followed by incubation at 37 °C for 7 h. Afterwards, the bacterial cells were harvested by centrifugation at 5000 rpm for 5 min at 4 °C. Lysis buffer was added at 1:10 (*w*/*v*, cell pellet weight to buffer volume), and the bacterial cells were sonicated at 25% power for 30 min (Scientz Biotechnology Co., Ltd., Ningbo, Zhejiang, China). The supernatant and precipitate were separated, and the latter was redissolved in equilibration buffer, followed by an additional 15 min of sonication and centrifugation, and the supernatant was purified using a Ni-NTA affinity column (Tiandirenhe Biotechnology Co., Ltd., Changzhou, Jiangsu, China). The purified eluate was subjected to SDS-PAGE and Western blot to verify protein expression. Finally, the eluted protein was subjected to stepwise gradient renaturation at 4 °C using renaturation buffer with a descending urea concentration gradient, followed by ultrafiltration and concentration to determine the protein concentration.

### 3.16. Experimental Verification of Multi-Epitope Subunit Vaccine

#### 3.16.1. Preclinical Proof-of-Concept for Vaccine Candidate Immunogenicity

The multi-epitope subunit vaccine was emulsified 1:1 (*v*/*v*) with Freund’s adjuvant (complete/incomplete) (Sigma-Aldrich, St. Louis, MO, USA) and administered to 6–8-week-old BALB/c mice (SPF (Beijing) Biotechnology Co., Ltd., Beijing, China) (*n* = 3 per group) according to Table 5 [52,53]. This immunization strategy is a standard method for evaluating vaccine candidates, ensuring robust immune activation and affinity maturation to maximize the immune response. Antibody titers following immunization are known to peak between 7 and 14 days after the final boost. Accordingly, to target this window of maximal response, tail vein blood was collected on day 10 after the fourth immunization for serum isolation [54,55]. All animal procedures were approved by the Institutional Animal Care and Use Committee (IACUC) of China Agricultural University (Approval No. AW12705202-1-01) and were conducted in compliance with the relevant animal welfare guidelines. Serum titers were quantified via indirect ELISA [56]: (1) Coating: Antigen coating concentration was optimized by checkerboard assay (two-fold serial dilutions, 100 μL/well, 4 °C overnight). (2) Blocking: Washed plates (PBST ×5) were blocked with 5% skim milk/PBS (250 μL/well, 37 °C). (3) Incubation: Serum (serial dilutions, 100 μL/well) and negative controls (unimmunized mice) were incubated (37 °C, 1 h). (4) Detection: After washing, goat anti-mouse HRP-IgG (1:5000, 100 μL/well) (Jackson ImmunoResearch Laboratories, Inc., West Grove, PA, USA) was added. TMB substrate (100 μL/well) (Solarbio Science & Technology Co., Ltd., Beijing, China) was incubated (15 min, dark), reactions stopped with 50 μL stop solution, and OD450 was measured. (5) Analysis: Data (mean ± SEM) were plotted using Origin 2021.

#### 3.16.2. Preclinical Proof-of-Concept for Vaccine Candidate Cross-Reactivity

The multi-epitope vaccine candidate was separated by SDS-PAGE and transferred to PVDF membrane (100 mA, 90 min) [57]. After five TBST washes (5 min each), the membrane was blocked with 5% skim milk/TBST (2 h) [58,59]. Pooled positive serum from immunized ducks (containing DHAV-I QYD [GU066825.1] and DHAV-3 GY [EU352805.2]) was diluted 1:100 in blocking buffer and incubated overnight at 4 °C. Following washes, HRP-conjugated rabbit anti-duck IgY (1:5000) (biorbyt Ltd., Cambridge, UK) was added (1 h incubation). Protein bands were detected using ECL substrate (Beyotime Biotechnology Co., Ltd., Shanghai, China).

To quantitatively validate the cross-reactivity of the vaccine candidate, an indirect ELISA was performed using mouse immune serum against recombinant VP1 structural proteins. Briefly, the plates were coated with 15 μg/mL of either DHAV-1 VP1 (GenBank: QKX94978.1) or DHAV-3 VP1 (GenBank: AJ177108.1), which were prepared in our laboratory. Serum from immunized mice (exemplified by Mouse #1 serum) was subjected to serial dilution and assayed following the same procedure detailed in Section 3.16.1.

## 4. Conclusions

Although commercially approved DHAV vaccines are available, the protective efficacy of monovalent vaccines against multiple genotypes remains suboptimal. Subunit vaccines—comprising small peptides or proteins—exhibit low adverse effects and potent immunogenicity, enabling effective protection through rational design. In this study, a multi-epitope subunit vaccine candidate was designed using immunoinformatics approaches by selecting 18 key antigenic epitopes across different DHAV genotypes. Computational analyses confirmed that the candidate exhibits high antigenicity, epitope conservancy, favorable stability, and effective binding affinity to duck Toll-like receptor 7 (TLR7), a key innate immune receptor. Preliminary experimental validation demonstrated that the candidate vaccine induced robust antibody responses in mice, with titers reaching up to 1:128,000. Crucially, the immune sera showed significant cross-reactivity with recombinant VP1 proteins of both DHAV-1 and DHAV-3, and the vaccine antigen was recognized by sera from ducks co-infected with DHAV-1 and DHAV-3. Collectively, these findings indicate that our constructed vaccine candidate can elicit broad immunorecognition against major prevalent genotypes. This work provides a promising strategy and solid preclinical evidence for developing a broad-spectrum vaccine capable of overcoming intergenotypic differences in DHAV, laying a foundation for subsequent clinical evaluation. Moreover, the integrated platform of computational design and preliminary experimental validation established in this study offers a scalable framework for the rapid development of multivalent poultry vaccines.

## Figures and Tables

**Table 1:** Selected DHAV proteins used for the multi-epitope vaccine candidate design.

Protein Name	Uniprot/NCBI Sequence lD	Antigenicity Score
Avihepatovirus A (VP1)	ACD68196.1	0.4105
Avihepatovirus A (VP1)	ACD68195.1	0.4183
Avihepatovirus A (VP3)	ACC78155.1	0.4731
Avihepatovirus A (VP3)	ACD68191.1	0.4768
Avihepatovirus A (VP0)	ACD68185.1	0.4746
Avihepatovirus A (VP0)	ACC78146.1	0.5113

**Table 2:** Selected B-cell epitopes showing antigenicity and toxin.

S. No.	Epitope	Antigenicity Score	Toxin
1	EPVCFLN	1.4923	Non-Toxin
2	VPLVRTVQHASTVQELD	0.7518	Non-Toxin
3	SRLVHLVTGQ	0.8328	Non-Toxin
4	CLRWLATPV	0.6351	Non-Toxin
5	AICVIVLGK	0.6243	Non-Toxin
6	IADGEQS	0.9743	Non-Toxin
7	YGNLQMAT	0.6972	Non-Toxin

**Table 3:** Selected HTL epitopes showing antigenicity and toxin.

S. No.	Epitope	Antigenicity Score	Toxin
1	IILTIVNNGTTPAMV	0.4587	Non-Toxin
2	SLSVFMGLKKPALFF	0.4362	Non-Toxin
3	GYFRFCLRLKTLAFE	1.3617	Non-Toxin
4	PYGYLMWHVVNRLTV	0.4496	Non-Toxin
5	IMVLRRWQILASFQW	0.8046	Non-Toxin

**Table 4:** Selected CTL epitopes showing antigenicity and toxin.

S. No.	Epitope	Antigenicity Score	Toxin
1	VERRSLMNL	0.6863	Non-Toxin
2	TEIDLVVPY	0.7126	Non-Toxin
3	AMVAHSYSM	0.4858	Non-Toxin
4	YQMSWYPIA	1.5676	Non-Toxin
5	SEYAVTAMG	0.6279	Non-Toxin
6	KDFQFTAPL	1.3095	Non-Toxin

**Table 5:** The immunization protocol.

Experimental Animals	Days	Frequency of Immunization	Immunization Dose (μg/Animal)	Adjuvant	Immunization Sites
BALB/c Mouse	0	Initially	50	FCA	Subcutaneous area of the nape and back of the neck
	21	Secondly	50	FIA	
	35	Thirdly	50	FIA	
	49	Fourthly	50	FIA	

## Data Availability

Data will be made available on request.

## Supplementary Materials

The following supporting information can be downloaded at: https://www.mdpi.com/article/10.3390/ijms262210958/s1.
