# Supplementary material for: A Highly Immunogenic and Cross-Reactive Multi-Epitope Vaccine Candidate Against Duck Hepatitis A Virus: Immunoinformatics Design and Preliminary Experimental Validation
Source: Int J Mol Sci. 2025 Nov 12;26(22):10958. doi: 10.3390/ijms262210958 (PMC12652605; doi:10.3390/ijms262210958)
Supplement: Supplementary file 1 [file ijms-26-10958-s001.zip › ijms-3902424-supplementary.pdf]

Relevant binding parameters

HADDOCK score	-111.0 +/- 21.2
Cluster size	2
RMSD from the overall lowest-energy structure	8.9 +/- 0.2
Van der Waals energy	-48.7 +/- 8.6
Electrostatic energy	-265.4 +/- 51.6
Desolvation energy	-9.2 +/- 2.3
Restraints violation energy	0.0 +/- 0.0
Buried Surface Area	2225.9 +/- 307.4
Z-Score	-1.8

Table S1 Conservancy Analysis of Key Epitopes with High Conservation Rates.

No.	Epitope Sequence	Protein Source	Target Genotype	Conservancy (%)
1	SEYAVTAMG	VP1	DHAV-1	96.58
2	SEYAVTAMG	VP1	DHAV-3	95.36
3	SEYAVTAMG	VP3	DHAV-3	91.95
4	AMVAHSYSM	VP1	DHAV-1	93.84
5	SLSVFMGLKKPALFF	VP1	DHAV-1	87.67
6	IILTIVNNGTTPAMV	VP1	DHAV-1	71.92
7	EPVCFLN	VP1	DHAV-3	94.94
8	EPVCFLN	VP3	DHAV-3	96.55

Table S2 Serum antibody titer in mice immunized with the multi-epitope vaccine candidate.

Animal number	Serum dilution factor										
	Positive										Negative
	1:500	1:1000	1:2000	1:4000	1:8000	1:16000	1:32000	1:64000	1:128000	1:256000	1:500
mouse1	3.506	3.428	3.383	3.306	3.375	2.564	1.619	0.923	0.515	0.289	0.127
	3.626	3.383	3.384	3.436	3.15	2.44	1.491	0.812	0.507	0.274	0.103
mouse2	3.394	3.283	3.408	3.247	2.675	1.708	1.085	0.631	0.35	0.213	0.112
	3.399	3.288	3.351	3.322	2.359	1.709	1.062	0.568	0.329	0.206	0.098
mouse3	3.337	3.37	3.264	3.078	2.452	1.641	0.991	0.491	0.309	0.198	0.109
	3.417	3.418	3.279	3.222	2.418	1.447	1.006	0.563	0.323	0.191	0.107

Recombinant VP Protein Information

DHAV1-VP1 sequence: (GenBank: QKX94978.1)

GDSNQLGDDEPVCFLNFETANVPIQGESHTLVKHLFGRQWLVMTVQHASTVQ
ELDLQVPDRGHASLIRFFAYFSGEIILTIVNNGTTPAMVAHSYSMDDLSSEYAVT
AMGGVMIPANSAKNISVPFYSVTPLRPTRPIPGTSEATFGRLFMWTQSGSLSVF
MGLKKPAFFFPLPAPTSTILSQRSNDVIPTLNQSRDEVDCHFCEICSKM
KRRWKPRGYSRFCLRLKTLAFELNLEIE

DHAV3-VP1 sequence (GenBank: AJ177108.1)

GDSNQLGDDEPVCFLNFETANVPIQGESHTLVKHLFGRQWLVRTVQHTSEVQ
ELDLPVPDQGHASLLRFFAYFSGEVILTIVNNGTTPCMVAHSYTMDNLTSEYAV
TAMGGILIPANSAKNINIPFYSVTPLRPTRPMPTFQGGGLTFGRLYIWTQSGSVS
VFMGLHKPALFFPLPAPTYTTHTQLNNIETMNLHNQSDQPDCHLCKICK
KMKKWSRNHRPFRFCLRLKTLAFELHLEIE

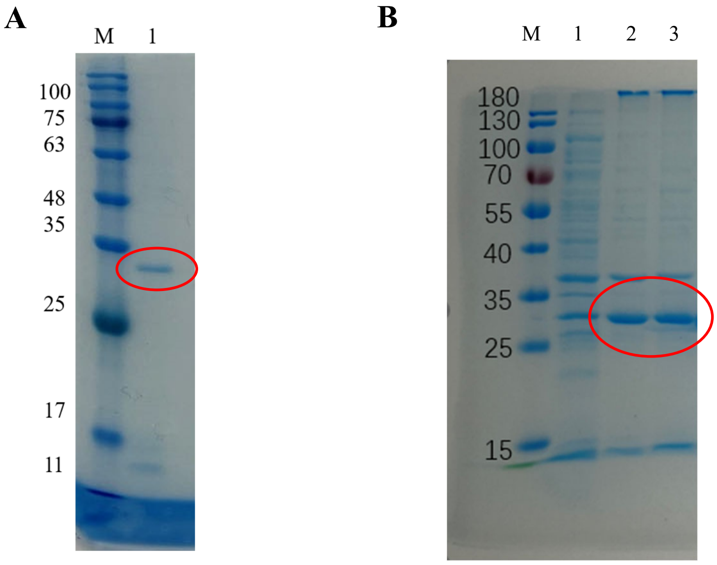

DHAV1-VP1 DHAV3-VP1

Table S3 Cross-reactivity of serum from mouse 1 immunized with the multi-epitope vaccine candidate against both DHAV-1 VP1 and DHAV-3 VP1

Coating Antigen	Serum dilution factor							
	Positive							Negative
	1:500	1:1000	1:2000	1:4000	1:8000	1:16000	1:32000	1:500
DHAV-1 VP1	2.879	2.793	2.534	1.776	1.011	0.637	0.377	0.105
	2.861	2.797	2.511	1.702	1.092	0.652	0.356	0.115
DHAV-3 VP1	2.789	2.441	1.593	0.976	0.559	0.37	0.243	0.118
	2.761	2.432	1.612	0.98	0.601	0.374	0.241	0.12
